# P-2164. Diagnostic Utility of Plasma Microbial Cell-free DNA Sequencing in Suspected Infective Endocarditis and the Impact on Treatment Decisions

**DOI:** 10.1093/ofid/ofae631.2318

**Published:** 2025-01-29

**Authors:** Myeongji Kim, Pansachee Damronglerd, Nischal Ranganath, Zachary Yetmar, Omar M Abu Saleh, Maryam Mahmood

**Affiliations:** Mayo Clinic, Rochester, Minnesota; Faculty of Medicine Thammasat University, Rochester, Minnesota; Mayo Clinic, Rochester, Minnesota; Cleveland Clinic, Cleveland, Ohio; Mayo Clinic Rochester, Rochester, Minnesota; Mayo Clinic, Rochester, Minnesota

## Abstract

**Background:**

Plasma microbial cell-free DNA (mcfDNA) sequencing, commercially available from Karius^®^ (Redwood City, CA), has been increasingly utilized in diagnosing endovascular infections. The 2023 updated Duke criteria incorporated detection of pathogen via metagenomic sequencing in its microbiologic criteria. We analyzed the clinical impact of mcfDNA sequencing in the diagnosis and treatment of suspected infective endocarditis (IE) cases.

**Figure:**
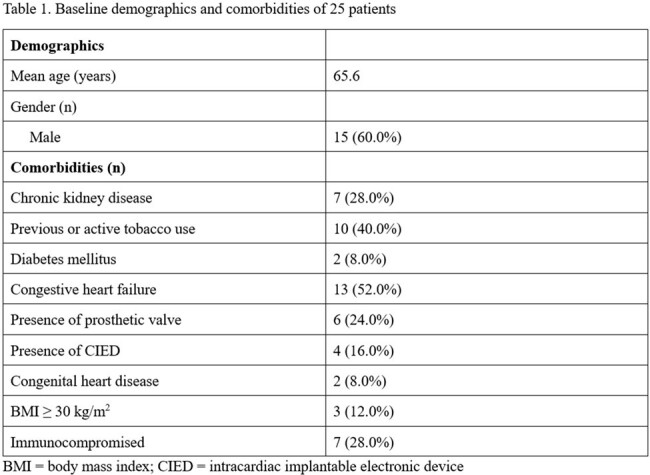

**Methods:**

We performed retrospective review of patients with suspected IE who underwent Karius^®^ test at Mayo Clinic, MN, between 2017 to 2023.

**Figure:**
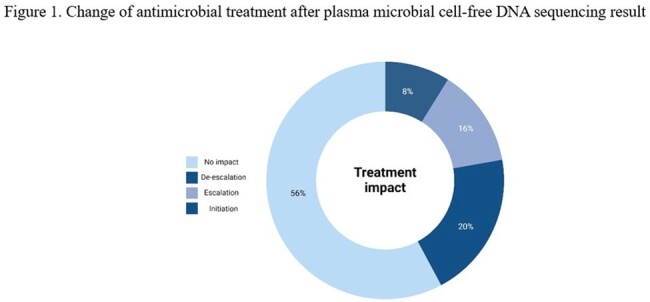

**Results:**

We preformed preliminary analysis of 25 patients. Analysis of baseline characteristics (table 1) revealed that these patients had significant predisposing factors for IE. Before accounting for mcfDNA sequencing, 3 (12%) met criteria for definite IE, 10 (40%) met criteria for possible IE, and 12 (48%) were rejected per 2023 Duke criteria. After incorporating mcfDNA sequencing result, 1 patient who was classified as possible IE was escalated to definite IE. This patient had previous infection of implantable cardioverter-defibrillator (ICD) with *Streptococcus dysgalactiae*. ICD was removed. She had development of endophthalmitis and stroke, 1 week and 2 weeks after the completion of IV antibiotics, respectively, with echocardiogram showing new tricuspid valve vegetation. Routine work-up was negative despite that she was not on any systemic antibiotics at the time of blood culture collection. mcfDNA sequencing detected *Streptococcus dysgalactiae*, making definite diagnosis of IE, leading to the physician’s decision to extend the IV antibiotic treatment (treatment escalation). Overall, after mcfDNA sequencing result, treatment decision was not impacted in 14 (56%), ongoing treatment was escalated in 4 (16%), de-escalated in 5 (20%), and antimicrobials was newly initiated in 2 (8%) (figure 1). Positive mcfDNA sequencing result often led to treatment escalation or initiation; negative result often led to treatment de-escalation (figure 2).

**Figure:**
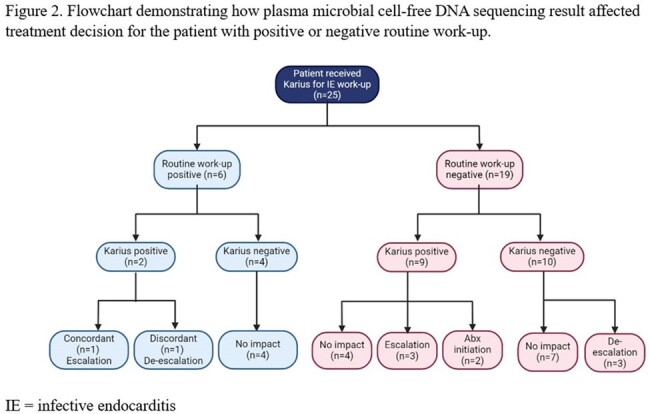

**Conclusion:**

In challenging cases of suspected IE, mcfDNA sequencing led to alteration of antimicrobial treatment in 44% of cases. Further data collection and final analysis will include larger number of patients.

**Disclosures:**

All Authors: No reported disclosures

